# A Potential Target for Clinical Atherosclerosis: A Novel Insight Derived from TPM2

**DOI:** 10.14336/AD.2021.0926

**Published:** 2022-04-01

**Authors:** Ling-bing Meng, Hong-xuan Xu, Meng-jie Shan, Gai-feng Hu, Long-teng Liu, Yu-hui Chen, Yun-qing Liu, Li Wang, Zuoguan Chen, Yong-jun Li, Tao Gong, De-ping Liu

**Affiliations:** ^1^Department of Cardiology, Beijing Hospital, National Center of Gerontology, Institute of Geriatric Medicine, Chinese Academy of Medical Sciences, Beijing, China.; ^2^Graduate School, Chinese Academy of Medical Sciences & Peking Union Medical College, Beijing, China.; ^3^Department of plastic surgery, Peking Union Medical College Hospital, Beijing, 100730, China.; ^4^Department of Cardiology, The First A?liated Hospital of Wenzhou Medical University, Wenzhou, China.; ^5^Department of pathology, Beijing Hospital, National Center of Gerontology, National Center of Gerontology, Institute of Geriatric Medicine, Chinese Academy of Medical Sciences, Beijing, China.; ^6^Department of neurology, Beijing Hospital, National Center of Gerontology, National Center of Gerontology, Institute of Geriatric Medicine, Chinese Academy of Medical Sciences, Beijing, China.; ^7^Department of Vascular Surgery, Beijing Hospital, National Center of Gerontology, National Center of Gerontology, Institute of Geriatric Medicine, Chinese Academy of Medical Sciences, Beijing, China.

**Keywords:** Atherosclerosis, tropomyosin 2, cardio-cerebrovascular diseases, clinical sample, artery

## Abstract

Atherosclerosis (AS) is a potential inducer of numerous cardio-cerebrovascular diseases. However, little research has investigated the expression of TPM2 in human atherosclerosis samples. A total of 34 clinical samples were obtained, including 17 atherosclerosis and 17 normal artery samples, between January 2018 and April 2021. Bioinformatics analysis was applied to explore the potential role of TPM2 in atherosclerosis. Immunohistochemistry, immunofluorescence, and western blotting assays were used to detect the expression of TPM2 and α-SMA proteins. The mRNA expression levels of TPM2 and α-SMA were detected using RT-qPCR. A neural network and intima-media thickness model were constructed. A strong relationship existed between the intima-media thickness and relative protein expression of TPM2 (P<0.001, R=-0.579). The expression of TPM2 was lower in atherosclerosis than normal artery (P<0.05). Univariate logistic regression showed that TPM2 (OR=0.150, 95% CI: 0.026-0.868, P=0.034) had clear correlations with atherosclerosis. A neural network model was successfully constructed with a relativity of 0.94434. TPM2 might be an independent protective factor for arteries, and one novel biomarker of atherosclerosis.

Atherosclerosis (AS) is a chronic disease of artery walls and a leading cause of death and life-year loss worldwide [1,2]. It is a chronic inflammatory disease characterized by lipid deposition and plaque formation caused by multiple injuries, which can lead to a variety of cardiovascular and cerebrovascular diseases, such AS stroke, coronary atherosclerotic heart disease, and myocardial infarction [2,3]. It is generally believed that acute cardiovascular and cerebrovascular events are caused by the rupture of unstable plaques and blockage of blood vessels by thrombosis[4]. The main pathological process is endothelial cell injury, while macrophage phagocytosis of oxidized low-density lipoprotein (ox-LDL) cholesterol turns them into foam cells that accumulate subcutaneously in the arterial wall and form lipid plaques [5,6]. However, the molecular mechanisms underlying the formation and rupture of unstable plaques are not fully understood.

TPM2 is a protein-coding gene, a subtype of the TPM family, which has the function of stabilizing and integrating actin filaments [7]. The associated pathways include cardiac conduction and dilated cardiomyopathy (DCM). In our previous research, tropomyosin 2 (TPM2) was reported as a potential predictive biomarker for atherosclerosis based on the screening of bioinformatics analysis and the verification of animal experiment. Compared with the non-atherosclerotic tissues, the expression of TPM2 was down-regulated in the atherosclerosis samples. Through the Gene Ontology (GO) and Kyoto Encyclopedia of Genes and Genomes (KEGG) analysis, differently expressed genes (including TPM2) between non-atherosclerosis and atherosclerosis were primarily enriched in actin filament, actin binding, smooth muscle cells, and cytokine-cytokine receptor interactions. TPM2 might be a potential novel atherosclerosis suppressor gene in vascular tissues, and it might represent a promising therapeutic gene target for atherosclerotic patients [7]. However, no studies have yet confirmed whether the expression level of TPM2 in human AS tissues is lower than that of non-atherosclerosis tissues.

**Figure 1.**
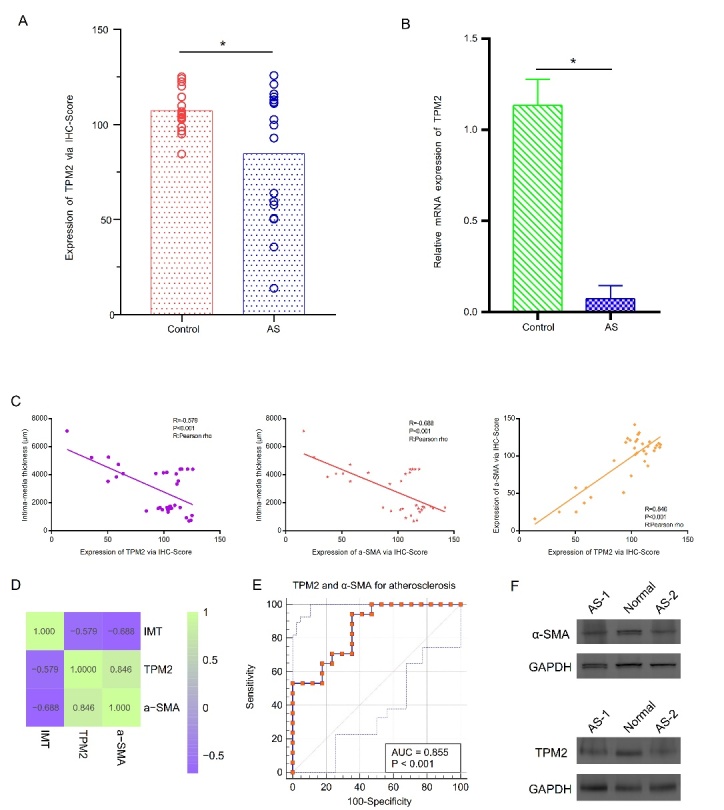
Expression level of TPM2 via immunohistochemistry, immunofluorescence, and RT-qPCR; correlation analysis of the intima-media thickness, TPM2, and α-SMA; and expression of TPM2 and α-SMA at the western blotting level. (A) A comparison of the H-scores of TPM2 in the two groups. (B) Relative expression of TPM2 by RT-qPCR analysis. (C) Correlation analysis of the intima-media thickness, TPM2, and α-SMA. (D) A heatmap showing the strong correlations among the intima-media thickness, TPM2, and α-SMA. (E) ROC curves were constructed to determine the effect of TPM2 and α-SMA on the intima-media thickness. (F) Expression of TPM2 and α-SMA at the western blotting level. *P< 0.05, compared with the normal group.

Therefore, the aim of this study was to explore whether the expression of the TPM2 gene in human AS tissues is lower than that in non-atherosclerotic vessels.

## Lower expression of TPM2 in the clinical atherosclerosis samples, and strong correlation of the intima-media thickness, TPM2, and α-SMA

AS is the primary basis for most cardiovascular diseases, including myocardial infarction and stroke [8]. The immunohistochemistry and immunofluorescence assays showed that the expression of TPM2 protein in the atherosclerosis group was lower than that in the normal group (P<0.05, Fig. 1A). These results indicated that the relative expression level of TPM2 mRNA was significantly increased in artery tissue samples without atherosclerosis compared with artery tissue samples with atherosclerosis (P<0.05, Fig. 1B).

Pearson rho analysis showed that a strong relationship existed between the intima-media thickness and relative protein expression of TPM2 (P<0.001, R=-0.579). The intima-media thickness was also related to the relative protein expression of α-SMA (P<0.001, R=-0.688). In addition, a strong relationship existed between α-SMA and the relative protein expression of TPM2 (P<0.001, R=0.846, Fig. 1C). A heatmap showed that there were strong correlations among the intima-media thickness, TPM2, and α-SMA (Fig. 1D). ROC curves were constructed to determine the effect of TPM2 and α-SMA on the intima-media thickness, with the degree of confidence judged by the area under the curve (AUC)=0.855 (P<0.001, Fig. 1E). Western blotting analysis showed that the expression of α-SMA and TPM2 proteins was lower in the atherosclerosis group than in the normal group (Fig. 1F). Tropomyosins (TPMs) are a family of actin-binding proteins whose expression is highly tissue-specific. The role of TPMs is to stabilize and integrate actin filaments. In addition, TPMs are related to cell migration and morphological changes. TPMs are widely distributed in various eukaryotic cells in the form of a large number of isomers. Four TPM gene subtypes, TPM1, TPM2, TPM3, and TPM4, have been identified in mammals [9]. Lin et al. showed that Mir-183-5 p.1 inactivates the Bcl-2/P53 signaling pathway and promotes cell proliferation, migration, and invasion by down-regulating TPM1 [10]. Cui et al. found that TPM2 is often silenced following abnormal DNA methylation, while the loss of TPM2 is associated with RhoA activation and cell proliferation [11]. Therefore, we hypothesized that TPM2 may be related to cell proliferation and migration during the development of atherosclerosis.

**Table 1:** Correlative parameters’ effect on atherosclerosis based on univariate logistic proportional regression analysis

Parameters			Atherosclerosis		
			OR	95% CI	P
Sex	Male	27	1		0.672
	Female	7	0.696	0.130-3.724	
Age	<60	4	1		1.000
	≥60	30	1.000	0.124-8.057	
Diabetes	No	25	1		0.251
	Yes	9	2.545	0.516-12.546	
Hypertension	No	11	1		0.276
	Yes	23	2.275	0.518-9.989	
Smoking	No	31	1		0.553
	Yes	3	2.133	0.175-26.033	
Hematencephalon	No	30	1		0.309
	Yes	4	0.292	0.027-3.133	
Cerebral infarction	No	19	1		0.730
	Yes	15	1.270	0.327-4.930	
CHD	No	24	1		0.454
	Yes	10	1.773	0.396-7.932	
TPM2	Low	10	1		0.034*
	High	24	0.150	0.026-0.868	
α-SMA	Low	12	1		0.038*
	High	22	0.190	0.040-0.915	

OR, odds ratio; 95% CI, 95% confidence interval. CHD: Coronary heart disease; TPM2: Tropomyosin 2; α-SMA: α-smooth muscle actin. *P<0.05.

**Figure 2.**
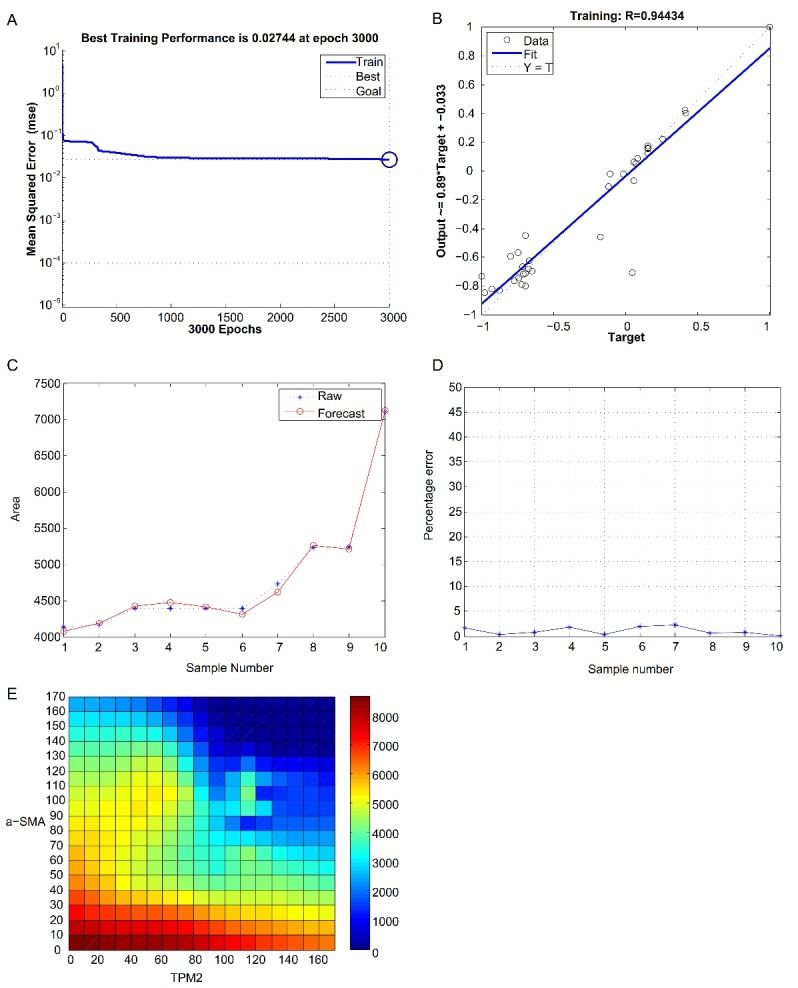
The neural network model for prediction of the intima-media thickness and thin-plate spline interpolation. (A) The best training performance was 0.02744 at epoch 3000. (B) The final training model of the neural network prediction model, with a relativity of 0.94434. (C, D) The model verified the result, and there were no significant differences between the predicted and actual values. (E) Using thin-plate spline interpolation, a high-risk warning indicator of the intima-media thickness was developed: 0 < TPM2 < 80, and 0 < α-SMA < 40.

## Protective effect of high expression level of α-SMA and TPM2 for atherosclerosis via logistic regression and neural network model

Table 1 shows the odds ratios (ORs) and 95% confidence interval (CI) of the individuals using univariate logistic regression; it can be seen that TPM2 (OR=0.150, 95% CI: 0.026-0.868, P=0.034) and α-SMA (OR=0.190, 95%CI: 0.040-0.915, P=0.038) have clear correlations with atherosclerosis. However, there were no significant correlations between atherosclerosis and between sex, age, diabetes, hypertension, smoking, drinking alcohol, hematencephalon, cerebral infarction, or coronary heart disease. After training of 3000 steps in the neural network model, the best training performance was 0.02744 at epoch 3000 (Fig. 2A), and the relativity was 0.94434 (Fig. 2B). Then, the model was used to verify the results and showed there were no significant differences between the predicted and actual values (Fig. 2C, D). Based on the above results, we speculate that the levels of TPM2 and α-SMA expression might be predictive indexes for intima-media thickness. Using the thin-plate spline interpolation, we found a high-risk warning indicator of the intima-media thickness: 0 < TPM2 < 80, and 0 < α-SMA < 40 (Fig. 2E). The development of atherosclerosis is a complex process in which cellular components or cytokines in vascular smooth muscle cells (VSMCs), endothelial cells, and monocytes/macrophages play an important role [12]. In recent years, studies have shown that smooth muscle cells (SMCs) account for about 70% of the cells contained in AS plaques. Initially, SMC proliferation, migration, and synthesis of extracellular matrix leads to early damage in AS. Next, pro-inflammatory and pro-proliferative cytokines, secreted by SMCs, activate SMCs and recruit macrophages to the damaged sites. Finally, SMCs absorb lipids through lipoprotein receptors expressed on their cell membrane surfaces and form myogenic foam cells [13-15].

More importantly, SMCs cannot function without regulation by the TPM2 gene. Previous studies have shown that senescence in VSMCs can promote further development of atherosclerosis in atherosclerotic tissues, suggesting that the normal function of VSMCs is crucial for protecting vascular walls against atherosclerosis [16]. Traditionally, the SMCs of the endometrium are considered beneficial for the formation of fibrous caps that prevent plaques from rupturing. Bennet et al suggested that VSMC proliferation may be beneficial throughout atherosclerosis [14]. Thus, during the development of atherosclerosis, the down-regulated expression of TPM2 leads to formation and movement disorders of VSMCs. The low expression of TPM2 in the tissues of patients with AS may accelerate the apoptosis of VSMCs, which leads to the release of various inflammatory factors, such as IL-1 and IL-8, aggravating the inflammatory response in the plaque, increasing atheromatous substances in the lipid nucleus, and making the plaque more unstable. Meanwhile, the apoptosis of VSMCs reduces anticoagulant components and promotes the rapid exposure of the anticoagulant phosphatidylserine, thus promoting the formation of thrombus [17]. In addition, apoptotic VSMCs are not easily removed, and the remaining part becomes the main component of the calcification matrix, leading to vascular calcification [18]. This is followed by weakened vasoconstriction, which further accelerates the occurrence of cardiovascular and cerebrovascular diseases.

## Limitations and future directions

Although this study has carried out a rigorous experimental design, there are still some deficiencies. There may be some selection bias in this paper. When conducting the research, the individuals in the study came from the one hospital, so selection bias will inevitably occur in the results. Due to the samples size is small, hypertension and smoking seemed not to be the risk factor for atherosclerosis when analyzing the relationship between characteristics of patients and atherosclerosis. However, sample collection was difficult because the number of surgeries for blood vessel has plummeted in this current pandemic of COVID-19. Furthermore, this research mainly focused on the significant role of TPM2 on the atherosclerosis. TPM2 might be a useful biomarker for the diagnosis and treatment of clinical atherosclerosis. Hypertension and smoking were only presented as traditionally basic characteristics of the patients, so that their value was not emphasized in this study. Therefore, in future studies, we will conduct a multi-center controlled clinical trial to collect more samples, and further research the relationships among TPM2, hypertension, smoking, and atherosclerosis.

## Conclusions

TPM2 was found to occur expressed at a low level in atherosclerotic plaques in patients. It was further found that TPM2 may be a useful biomarker for the diagnosis and treatment of atherosclerosis.

## Supplementary Materials

The Supplemenantry data can be found online at: www.aginganddisease.org/EN/10.14336/AD.2021.0926.


