# Supplementary material for: A Potential Target for Clinical Atherosclerosis: A Novel Insight Derived from TPM2
Source: Aging Dis. 2022 Apr 1;13(2):373–8. doi: 10.14336/AD.2021.0926 (PMC8947840; doi:10.14336/AD.2021.0926)
Supplement: Supplementary file 1 [file AD-13-2-373-s.pdf]

SUPPLEMENTARY DATA

A Potential Target for Clinical Atherosclerosis: A Novel Insight Derived from TPM2

Ling-bing Meng[1,2,#], Hong-xuan Xu[1,#], Meng-jie Shan[2,3,#], Gai-feng Hu[4,#], Long-teng Liu[5], Yu-hui Chen[6], Yun-qing Liu[1], Li Wang[6], Zuoguan Chen[7*], Yong-jun Li[7*], Tao Gong[6*], De-ping Liu[1,2*]

© 2021. Meng LB et al. Published online at http://www.aginganddisease.org/EN/10.14336/AD.2021.0926

SUPPLEMENTARY DATA

MATERIALS & METHODS

AS datasets

In this study, we obtained the transcriptome expression profiles GSE43292 (GPL6244), GSE57691 (GPL10558), and GSE125771 (GPL17586) of AS from the Gene Expression Omnibus (GEO) database (http://www.ncbi.nlm.nih.gov/geo/) (Table S1). The R package "limma" was employed to perform probe summarization, merging, and background correction of GSE43292, GSE57691, and GSE125771.

Scoring the microenvironment

The ESTIMATE algorithm is mainly based on the gene set enrichment analysis (GSEA) of a single sample, using expression profile data to score stromal cells and immune cells, and then predict the content of these two types of cells. In this study, we used the ESTIMATE algorithm to score the arterial microenvironment. We then used the R package to draw a scatter plot of the microenvironment scores to show the relationship between the samples and the scores.

The relationship between TPM2 and microenvironment scores

The stromal, immune, and ESTIMATE scores were used as the basis for grouping genes identified by differential gene screening. The R package "limma" was employed to investigate the relationship between TPM2 and the microenvironment score. The diagnostic value of the relationship between microenvironment score and TPM2 was tested using a receiver operating characteristic (ROC) curve.

Weighted gene co-expression network analysis (WGCNA)

WGCNA of all genes was performed using the R package "WGCNA". For WGCNA, 72 AS samples and 42 normal samples were used to construct a co-expression network for all genes. An adjacency matrix was created using the samples, which was then transformed into a topological overlap matrix (TOM). Genes were divided into different gene modules using TOM-based difference measurements. The minimal gene module was > 100, and the threshold to merge similar modules was 0.1; these values were used to search for modules that play an important role in AS. At the same time, WGCNA was also used to predict interconnections among genes in a module, and the data were then imported into Cytoscape software to map the connections between genes. TPM2 was also analyzed using Gene Ontology (GO) analysis and the results were visualized using the BiNGO plug-in in Cytoscape. Cytoscape software can provide biological network analysis and two-dimensional (2D) visualization for biologists. The BiNGO plug-in is a tool for determining which GO categories are statistically overrepresented in a set of genes or a subgraph of a biological network. BiNGO maps the predominant functional themes of a given gene set on the GO hierarchy and outputs this mapping as a Cytoscape graph.

Functional enrichment analysis

GSEA is a computational method that can execute GO and KEGG (Kyoto Encyclopedia of Genes and Genomes) analysis of a complete genome. In our study, we grouped the samples according to the level of expression of TPM2, and the GO and KEGG analyses of the complete genome were performed using GSEA.

Single gene analysis

Single gene difference analysis was performed using the R package "limma".

Identification of TPM2 associated with AS

The comparative toxicogenomics database (CTD database, http://ctdbase.org/) can be used to predict the relationship(s) between genes/proteins and disease. In our study, the relationships between TPM2 and AS were analyzed using this database.

© 2021. Meng LB et al. Published online at http://www.aginganddisease.org/EN/10.14336/AD.2021.0926

SUPPLEMENTARY DATA

Prediction of TPM2 competing endogenous RNAs (ceRNAs)

TargetScan (http://www.targetscan.org/vert_72/), miRDB (http://mirdb.org/), and miRTarBase (http://mirtarbase.cuhk.edu.cn) are online databases that can be used to systematically identify microRNA(miRNA)–mRNA interaction networks. LncACTdb 2.0 (http://www.bio-bigdata.net/LncACTdb/index.html) can be used to identify miRNA–mRNA and long non-coding RNA (lncRNA)–miRNA interaction networks. We used the first three databases to predict the target miRNAs of TPM2 and find the common miRNAs, then we used LncACTdb 2.0 to predict the target lncRNAs.

Identification of candidate molecule drugs for TPM2

The connectivity map (CMap, https://portals.broadinstitute.org/cmap/) database can be used to connect genes and genomic information with human diseases and the drugs that are used to treat them. In this study, molecular drugs that can regulate TPM2 were searched for using the CMap database.

Evaluation of AS-infiltrating immune cells

CIBERSORT is a computational approach that can be used to characterize immune cells in samples profiled by microarray or RNA sequencing (RNA-Seq) and accurately estimate the immune-cell composition. The gene expression signature matrixes of 547 marker genes that could quantify and define 22 immune cell subtypes were downloaded from the CIBERSORT web portal (http://cibersort.stanford.edu/). In our study, the differences in the distribution and number of immune cells between AS and normal tissues were calculated using CIBERSORT ($P<0.05$). The analyses in this study were conducted using Perl and R software (version 3.5.3). All cases with a P-value < 0.05 by the CIBERSORT analysis were included in the subsequent analyses. The Wilcoxon test was employed to detect differential infiltrations of the 22 immune cell types. Correlations among different immune cells were tested using the R package “corrplot”.

Characteristics of patients

There were 34 samples obtained for this study, comprising 17 samples with atherosclerosis and 17 samples without atherosclerosis. The samples were from patients who received treatment in the Beijing Hospital between January 2018 and April 2021. All the patients underwent surgery in the vascular surgery department or the pathology department. There were 17 atherosclerosis tissue samples and 17 normal arterial wall tissue samples. The tissue samples were got from the same size of the vessel. And there existed strongly positive relationship between the intima-media thickness of the arterial wall and the size of the vessel based on the Pearson’s rho test ($R=0.836$, $P<0.001$). Basic information for all patients was carefully recorded.

Among the patients from whom the samples were obtained, the normal group comprised 13 (38.2%) males and 4 (11.8%) females, while the atherosclerosis group comprised 14 (41.2%) males and 3 (8.8%) females. In terms of age, the normal group included 2 (5.9%) samples from patients aged <60 years and 15 (44.1%) samples from patients aged ≥60 years, and the atherosclerosis group included 2 (5.9%) samples from patients aged <60 years and 15 (44.1%) samples from patients aged ≥60 years. There were no differences between the normal and atherosclerosis groups in terms of diabetes, hypertension, smoking, drinking, hematencephalon, cerebral infarction, coronary heart disease, hyperlipidemia, or obesity ($P>0.05$). The mean and standard deviation of intima-media thickness was 1429.418 ±351.403 μm in the normal group and 4393.531 ±875.318 μm in the atherosclerosis group ($P<0.001$). The relative expression H-score of α-SMA was 109.633 ±14.898 in the normal group and 79.822 ±41.750 in the atherosclerosis group ($P=0.009$) (Table S2).

Inclusion and exclusion criteria

The inclusion criteria for the experiment group were: aged 18 to 110 years; patients with atherosclerosis; patients who underwent surgery in the vascular surgery or the pathology department; consent had been obtained from the patient and their family.

© 2021. Meng LB et al. Published online at http://www.aginganddisease.org/EN/10.14336/AD.2021.0926

SUPPLEMENTARY DATA

The inclusion criteria for the control group were: aged 18 to 110 years; patients clinically diagnosed as needing amputation or an organ transplant, or patients who have had an arterial blood vessel transplant; individuals without atherosclerosis.

Exclusion criteria included: patients with poor wound healing or infection; patients with severely weakened cardiopulmonary function; and patients with high preoperative intracranial pressure, encephalocele, and intracranial infection.

Immunohistochemistry and immunofluorescence to detect TPM2

Paraffin sections were again made using the tissue samples, as described above. The paraffin sections were deparaffinized with water, sealed with hydrogen peroxide, then washed with double-distilled water. TPM2 was detected using immunohistochemistry following antigen retrieval. Anti-TPM2 monoclonal antibody (Proteintech_11038-1-AP, Rosemont, USA) was used to detect TPM2. The specific detection steps were performed using a VECTASTAIN Elite ABC Kit (Vector Laboratories, Burlingame, CA, USA) as follows, according to the manufacturer's instructions. First, antigen-fixed paraffin sections were washed two to three times with phosphate-buffered saline (PBS) (5 min per time) and blocked with 10% goat serum (TransGen Biotech, Beijing, China) at 37°C for 20 min. Second, the serum was removed using filter paper and YAP or TAZ Rabbit polyclonal antibody (Abcam, Cambridge, UK) was added dropwise, then the samples were incubated overnight at 4°C. Third, the sections were washed three times (5 min per time) with PBS and incubated with goat anti-rabbit monoclonal antibody at 37°C for 1 hour. Fourth, color development was performed with diaminobenzidine. Each paraffin section was photographed.

Expression levels of α-SMA and TPM2 by RT-qPCR

Total RNA was extracted from samples using an RNAiso Plus (TRIzol) kit (Thermo Fisher Scientific, Inc.) and reverse transcribed into cDNA. RT-qPCR was performed in a Light Cycler® 4800 System (Roche) with a specific set of primers for the amplification of expressed genes. The relative quantification units ($RQ=2^{-\Delta\Delta Ct}$, where Ct represents quantification cycle values) of each sample were calculated and presented as fold change in gene expression relative to the control group. Primers and sequences of TPM2, α-SMA, and GAPDH are shown in Table S3. GAPDH was used as an endogenous control.

Detection of α-SMA and TPM2 protein expression by western blotting

First, we washed the clinical tissue samples two to three times with PBS. We then added lysis buffer (Servicebio, G2002, Wuhan, China) to isolate total protein. Each lane was loaded with 80 µg protein, then 10% sodium dodecyl sulfate polyacrylamide gel electrophoresis (SDS-PAGE) was performed. Second, the protein was transferred to polyvinylidene fluoride (PVDF) membranes, and 5% skimmed milk was added for 1 hour to seal the membrane. Primary antibody was added, and the samples were incubated overnight at 4°C. Horseradish peroxidase-labeled secondary antibody was added, and the protein samples were incubated for 2 h at 25°C.

GAPDH was used as endogenous control. Mouse anti-rabbit GAPDH polyclonal antibody (dilution rate = 1:1000, GB12002, Servicebio, Wuhan, China) was used to detect GAPDH, while the secondary antibody was a goat anti-mouse monoclonal antibody (dilution rate = 1:5000, ab205719, Abcam, Cambridge, UK). α-SMA protein was detected using an α-SMA polyclonal antibody (dilution rate = 1:5000, 14395-1-AP, Proteintech, Rosemont, USA). TPM2 protein was detected using an TPM2 polyclonal antibody (dilution rate = 1:3000, 11038-1-AP, Proteintech, Rosemont, USA). A secondary antibody was applied (dilution rate = 1:5000). The films were scanned using an enhanced chemiluminescence kit (Millipore, Billerica, Massachusetts, USA). Image-Pro Plus 6.0 (Media Cybernetics Inc., Chicago, USA) was used to analyze the optical density values of the target bands.

Statistical analysis

The data were expressed as percentages of the totals and means ± SD. Fisher's test was used for categorical variables. For continuous variables, an independent-samples t-test was used; when equal variances were not assumed, the Brown–Forsythe test was performed. Pearson's rho test was used to analyze correlations among α-SMA, TPM2, and the intima-media thickness. The ROC analyses in this study were conducted using MedCalc software. The effect of correlated

parameters on atherosclerosis were constructed using univariate logistic proportional regression analysis. A neural network model was constructed using α-SMA, TPM2, and the intima-media thickness to explore the usefulness of α-SMA and TPM2 values for predicting the intima-media thickness. Before performing neural network modeling, the input and output of neural network need to be determined. In this experiment, the data of α-SMA and TPM2 were used as input vectors to conduct limited validation of the intima-media thickness risk determination model, which could already reflect the health status of the body to a large extent due to the relatively large impact of these characteristics. To fit the data and give the risk of intima-media thickness, the model only needs a corresponding output. In order to find the number of neurons with the best learning effect, the number of neurons in the hidden layer is set to 3 ~ 12, and the training accuracy is set to 0.1, respectively. Through the learning of intima-media thickness data, these set learning processes are observed. In order to facilitate observation, this paper introduces the learning effect as the object of investigation, that is, the training error is reversed. Therefore, the number of neurons was determined to be 9 in this paper. Finally, quantitative relationships among α-SMA, TPM2, and the intima-media thickness were inferred using thin-plate spline interpolation. All statistical analyses were conducted using SPSS software, version 24.0 (IBM Corp., Armonk, NY, USA) and MATLAB (R2014a, MathWorks, Inc., New Mexico, USA). A p-value < 0.05 was considered statistically significant.

RESULTS

Evaluation of microenvironment scores

In this study, stromal, immune, and estimated scores of the samples derived from the GEO datasets were evaluated and are shown in Figure S1A. The diagnostic value of the three microenvironment scores was tested using ROC curves. The area under the curve of the stromal score for atherosclerosis was 0.782 (P<0.001), for the immune score was 0.817 (P<0.001), and for the estimate score was 0.824 (P<0.001) (Supplementary Figure 1B). The expression of TPM2 was significantly down-regulated in the atherosclerosis group compared with its expression in the normal group for all three scores (Supplementary Figure 1C-E).

WGCNA

Network topology analysis was performed to identify the soft-thresholding power. The soft-thresholding power in the WGCNA was set to 6, which was the lowest power for the scale-free topology fit index of 0.9 (Supplementary Figure 2A). A hierarchical clustering tree of all genes was constructed, and nine important modules were generated (Figure S2B). The dendrogram and heatmap of genes showed that there were no significant differences in interactions among the different modules, demonstrating that there was a high degree of independence between these modules (Supplementary Figure 2C). TPM2 was in the turquoise module, with a correlation value with the status of AS of -0.54 (P<0.05, Supplementary Figure 2D). There was significant correlation between module membership and gene significance in the turquoise module (cor=0.77, P<0.05, Supplementary Figure 2E). The predicted interconnections of TPM2 in the turquoise module were identified using Cytoscape software (Supplementary Figure 2F).

Functional enrichment analysis

In this study, GO analyses of predicted genes associated with TPM2 were performed using the BiNGO plug-in; the enriched terms were associated with actin binding, myofibrils, contractile fibers, contractile fiber components, and costameres (Supplementary Figure 2G). We then grouped the samples according to the level of expression of TPM2 and GO and KEGG analyses of the complete genome were performed using GSEA. The enrichment terms were significantly related to muscle cell differentiation, smoothened signaling pathway, structural constituents of muscle, actin, vascular smooth muscle contraction, and apoptosis (Supplementary Figure 2H).

Single gene analysis

TPM2 was differentially expressed in the merged series, and its expression was lower in the atherosclerosis group than in the normal group (Supplementary Figure 3A). The association between TPM2 and atherosclerosis was analyzed using an ROC curve (AUC=0.785, P<0.001) (Supplementary Figure 3B). The CTD database showed that TPM2 is

© 2021. Meng LB et al. Published online at http://www.aginganddisease.org/EN/10.14336/AD.2021.0926

associated with atherosclerosis, as shown in Figure S3C. The ceRNAs that regulate TPM2 were screened out (Supplementary Figure 3D).

Identification of candidate molecule drugs for TPM2

Small molecule drugs for atherosclerosis that could target TPM2 were screened using the CMap database; the small molecule drugs emetine, sodium phenylbutyrate, zomepirac, phensuximide, guanadrel, and lobelanidine were filtered by the number of instances (n> 3) and P-values (<0.05), as shown in Table S4.

Evaluation of atherosclerosis-infiltrating immune cells

In the atherosclerosis-infiltrating immune cell matrix, the stacked histogram shows the proportion of immune cells in each sample (Supplementary Figure 3E). Correlations among 22 immune cells are shown in Supplementary Figure 3F. As shown in Supplementary Figure 3G, naive B cells, memory B cells, plasma cells, CD8 T cells, memory resting CD4 T cells, memory activated CD4 T cells, gamma delta T cells, activated NK cells, monocytes and macrophages M0 infiltration content were different (P<0.05).

Supplementary Table 1. A summary of AS microarray dataset from different GEO datasets

Series	Platform	Affymetrix GeneChip	Normal	AS
**GSE43292**	GPL6244	[HuGene-1_0-st] Affymetrix Human Gene 1.0 ST Array [transcript (gene) version]	32	32
**GSE57691**	GPL10558	Illumina HumanHT-12 V4.0 expression beadchip	10	0
**GSE125771**	GPL17586	[HTA-2_0] Affymetrix Human Transcriptome Array 2.0 [transcript (gene) version]	0	40

© 2021. Meng LB et al. Published online at http://www.aginganddisease.org/EN/10.14336/AD.2021.0926

SUPPLEMENTARY DATA

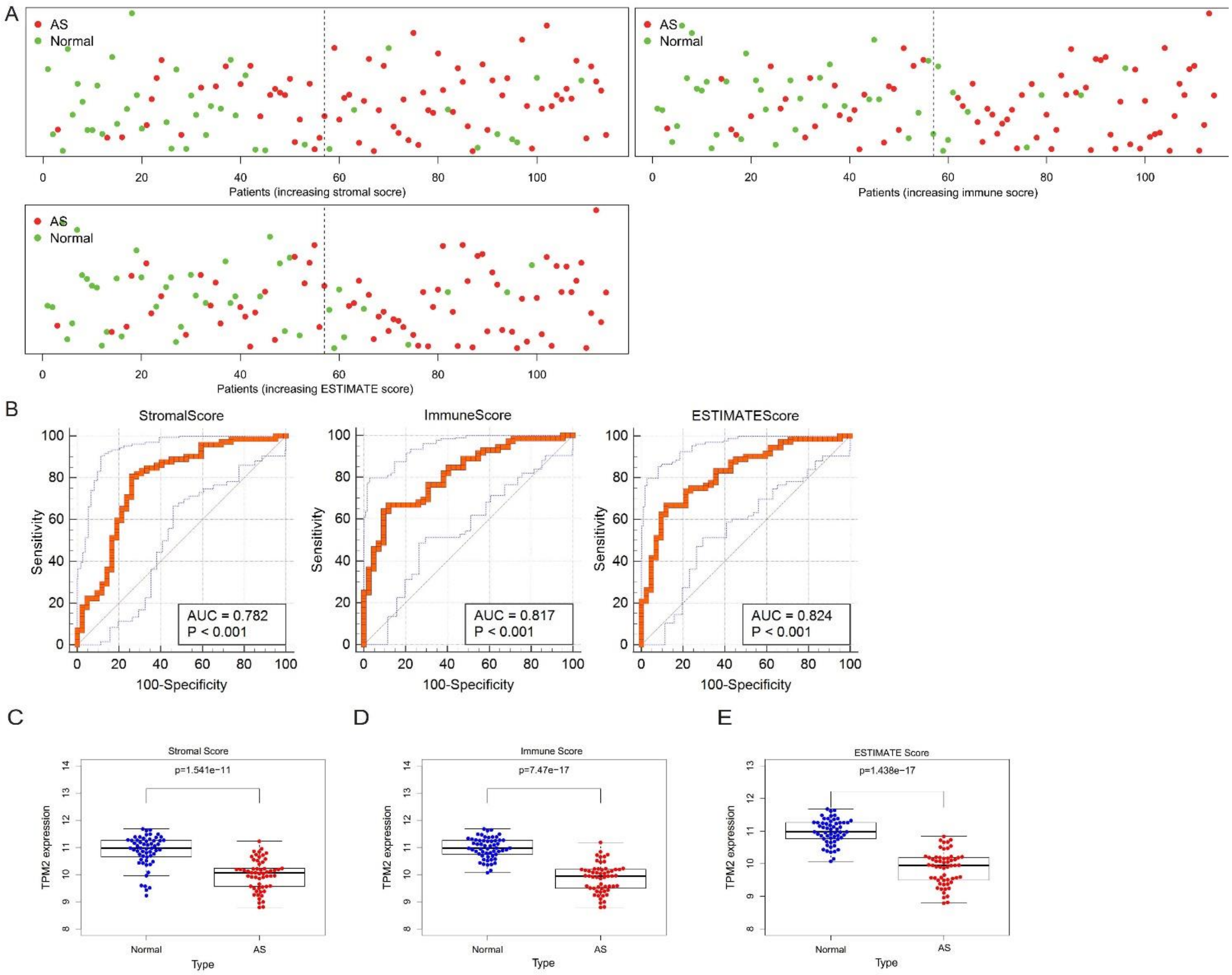

Supplementary Figure 1. The microenvironment in atherosclerosis. (A) Scatter plots showing the distribution of samples with microenvironment scores (stromal, immune, and ESTIMATE scores). The green dots represent people in the normal group, and the red dots represent patients with atherosclerosis. (B) The ROC curve shows the relationship between the microenvironment scores and atherosclerosis. (C-E) Box diagrams showing the differential expression of TPM2 by different scores.

© 2021. Meng LB et al. Published online at http://www.aginganddisease.org/EN/10.14336/AD.2021.0926

SUPPLEMENTARY DATA

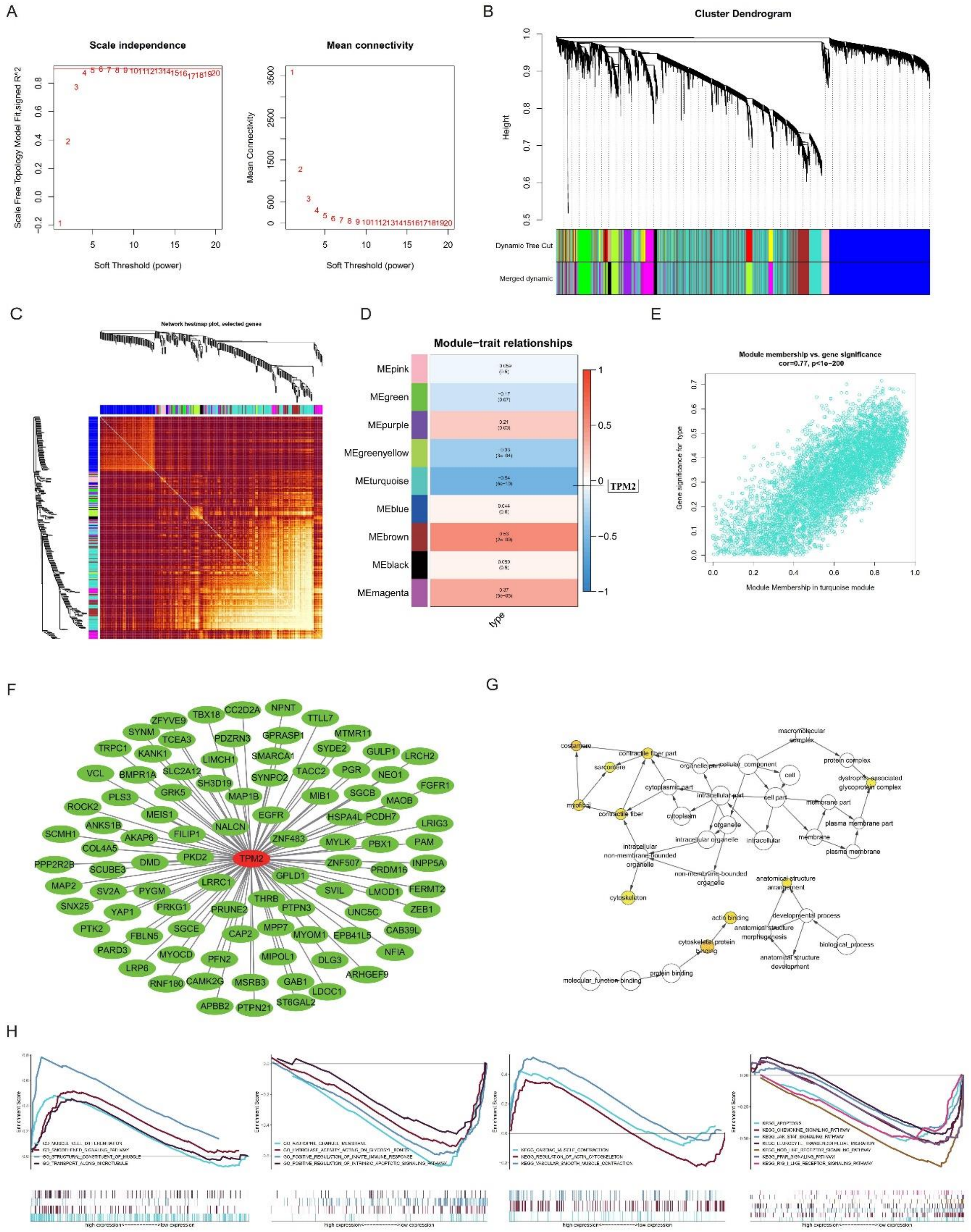

Supplementary Figure 2. WGCNA of TPM2. (A) The lowest power for which scale independence. (B) Repeated hierarchical clustering tree of all genes. (C) The dendrogram and heatmap of all genes. (D) The associations between atherosclerosis and the modules. (E) Module membership in the turquoise module. (F) Interrelationships between TPM2 and the genes in the turquoise module. (G) GO analysis of predicted genes associated with TPM2 in the turquoise module was constructed using the BiNGO plug-in in Cytoscape. (H) Gene functional enrichment analysis of the complete genome by GSEA, when the samples were grouped according to the level of TPM2 expression.

© 2021. Meng LB et al. Published online at http://www.aginganddisease.org/EN/10.14336/AD.2021.0926

SUPPLEMENTARY DATA

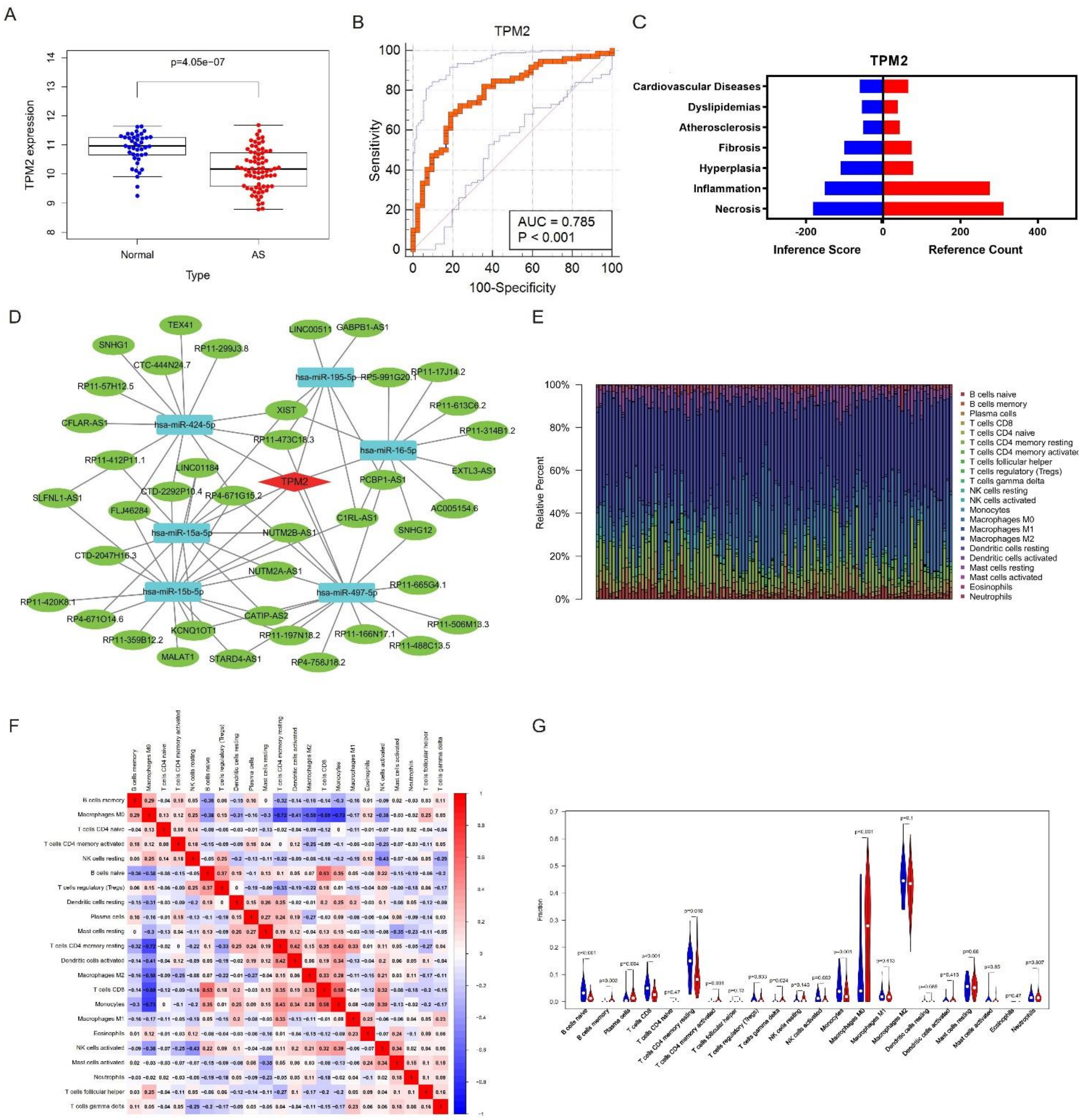

Supplementary Figure 3. Single gene analysis of TPM2 and atherosclerosis-infiltrating immune cells. (A) Differential expression analysis of TPM2. (B) The ROC curve shows the relationship between TPM2 and atherosclerosis. (C) Predicting the ceRNA network mechanism of TPM2. (D) Relationship between atherosclerosis and TPM2 based on the CTD database. (E) Fractions of immune cells in 42 normal and 72 atherosclerosis samples in the GEO database. (F) A correlational heatmap of 22 immune cells. (G) A violin plot of 22 immune cells found in atherosclerosis and normal tissues according to the GEO database; blue denotes normal tissue and red denotes atherosclerosis tissue.

© 2021. Meng LB et al. Published online at http://www.aginganddisease.org/EN/10.14336/AD.2021.0926

SUPPLEMENTARY DATA

Supplementary Table 2. Based characteristics of samples

Characteristics		Atherosclerosis		P
		No (%)	Yes (%)	
Sex				
Male	27	13(38.2%)	14(41.2%)	1.000
Female	7	4(11.8%)	3(8.8%)	
Age				
<60	4	2(5.9%)	2(5.9%)	1.000
≥60	30	15(44.1%)	15(44.1%)	
Diabetes				
No	25	14(41.2%)	11(32.4%)	0.438
Yes	9	3(8.8%)	6(17.6%)	
Hypertension				
No	11	7(20.6%)	4(11.8%)	0.465
Yes	23	10(29.4%)	13(38.2%)	
Smoking				
No	31	16(47.1%)	15(44.1%)	1.000
Yes	3	1(2.9%)	2(5.9%)	
Drinking				
No	33	17(50.0%)	16(47.1%)	1.000
Yes	1	0(0.0%)	1(2.9%)	
Hematencephalon				
No	30	14(41.2%)	16(47.1%)	0.601
Yes	4	3(8.8%)	1(2.9%)	
Cerebral infarction				
No	19	10(29.4%)	9(26.5%)	1.000
Yes	15	7(20.6%)	8(23.5%)	
CHD				
No	24	13(38.2%)	11(32.4%)	0.708
Yes	10	4(11.8%)	6(17.6%)	
Hyperlipidemia				
No	32	17 (50.0%)	15 (44.1%)	0.145
Yes	2	0 (0.0%)	2 (5.9%)	
Obesity				
No	32	17 (50.0%)	15 (44.1%)	0.145
Yes	2	0 (0.0%)	2 (5.9%)	
IMT	2911.475±1641.465	1429.418±351.403	4393.531±875.318	<0.001*
α-SMA	94.727±34.375	109.633±14.898	79.822±41.750	0.009*

Fisher test was used for categorical variable. For continuous variable, independent-samples T test was used, and when the equal variances not assumed, Brown-Forsythe was performed. CHD: Coronary heart disease. IMT: intima-media thickness; α-SMA: α-smooth muscle actin. *P<0.05.

Supplementary Table 3. Primers of GAPDH, TPM2, α-SMA and their sequences for PCR analysis

Primer	Sequence (5′–3′)
GAPDH-hF	TGAAGGTCGGAGTGAACGGAT
GAPDH-Hr	CGTTCTCAGCCTTGACCGTG
TPM2-hF	TCCACCAAGGAGGACAAATACG
TPM2-hR	GTTGTTGAGTTCCAGCAGGGTC
α-SMA-hF	ACCCTGTTGACTGAGGCACC
α-SMA-hR	ACCATCTCCAGAGTCCAGCAC

© 2021. Meng LB et al. Published online at http://www.aginganddisease.org/EN/10.14336/AD.2021.0926
